# Estimates of the potential risk of radiation-related cancer from screening in the UK

**DOI:** 10.1258/jms.2011.011073

**Published:** 2011-12

**Authors:** Amy Berrington de González

**Affiliations:** Radiation Epidemiology Branch, Division of Cancer Epidemiology & Genetics, NCI, Bethesda, MD, USA

Radiation exposure from medical imaging is one of the largest sources of radiation exposure to the general population, second after natural background exposures.^1^ In 2008 there were 46 million medical and dental examinations performed in the UK. The mean annual dose per person from this source increased by 23% between 1997 and 2008,^2^ primarily due to the doubling in the number of computed tomography (CT) scans over that period to 3.4 million per year. The average radiation dose from a CT scan is typically ten times higher than a conventional diagnostic X-ray (0.1–10 milli-Sievert [mSv] effective dose). There is concern about the potential risk of cancer from the increasing levels of medical radiation exposure in the UK and other developed countries. Furthermore, several types of CT scans, including lung CT, coronary artery calcification CT and CT colonography, have been proposed as new screening tools. The decision to expose large numbers of asymptomatic individuals to repeated radiation exposure raises legitimate concerns.

The Health Protection Agency's Advisory Group on Ionising Radiation commissioned a Sub-group on Solid Cancer Risk to write a report on cancer risks from radiation exposure to the UK population.^3^ The aims of the report were 1) to review information on the risk of solid cancers from exposure to ionising radiation, such as breast and lung cancer, but not on cancers such as lymphoma or leukaemia as these were reviewed in a separate report,^4^ 2) to derive risk estimates applicable to the UK population with a quantitative assessment of the effects of typical radiation exposures the public may experience. These risk estimates were then used to estimate the risk of radiation-related cancer for several types of screening examinations. The results of this exercise are summarized here along with a brief summary of the broader report.

There is a considerable amount of information on the risks of solid cancer from various epidemiological studies of radiation-exposed populations. The committee reviewed the available data to assess whether there is evidence of an association between cancers at specific sites and ionising radiation exposure and whether the association was likely to be causal. Assessment of causality was based on evidence of a dose-response relationship, the magnitude of the relative risk and the likelihood of uncontrolled confounding. For many, but not all solid cancers, the committee concluded that there was epidemiological evidence of an association with ionising radiation exposure and in most instances this association was judged to be causal; specifically, for cancers of the oesophagus, stomach, colon, rectum, liver, lung, bone, non-melanoma skin, breast (female), bladder and thyroid, together with brain and other central nervous system tumours. For cancers of the salivary glands and ovary, the association was judged to be probably causal. For some cancers it is unclear whether they are caused by ionising radiation exposure; these include cancers of the pancreas, connective tissue, melanoma of skin, uterine cervix, body of uterus, prostate and testis.

The lifetime risk of radiation-induced solid cancer was estimated for the UK population. These estimates were based on risk models developed using cancer incidence and mortality collected from the Japanese atomic bomb survivors Life Span Study.^5^ This study remains the basis for much risk assessment work due to its large size, long-term follow-up, and the wide variety of doses and range in age at exposure. However, data from studies of medically-exposed groups sometimes help to provide more pertinent estimates, particularly for rare cancers such as thyroid, bone and non-melanoma skin, and for breast cancer in females where baseline rates differ considerably between Japan and Western countries.

To conduct the calculations organ-specific radiation doses were estimated for each screening test from survey information^6^ or screening protocols^7–9^ and these were multiplied by the estimated lifetime risk of radiation-related cancer for each organ, for the relevant age at exposure and sex.^3^ The total cancer risk was then calculated by summing across all the exposed organs. There is debate about the most appropriate method to transfer risk models from the Japanese to other populations. The approach used in the report was to estimate risks using two models: 1) an *excess relative risk* model that assumes that the radiation exposure acts multiplicatively on the underlying cancer incidence rates and 2) an *absolute excess risk* model that assumes that the radiation exposure acts additively on the underlying rates. Results are presented from both models and to some extent these represent the range of likely risks. If there was a supra-multiplicative or sub-additive interaction then risks could lie outside this range but to date there is little evidence for such effects.

The risk estimates for repeated screening are summarized in Table 1. The smallest risks are for repeated mammography (0.3–0.6 cancers per 1000 women screened every three years from age 47–73). This is due to the relatively low radiation dose per mammographic X-ray and the fact that it only results in a measurable dose to one organ, the breast. The highest risk estimates were for lung CT screening in females (2.9–8.0 per 1000), primarily because annual screening was assumed due to the short pre-clinical detection period for lung cancer screening with CT.^10^ If individuals underwent all of these screening tests routinely then over the lifetime there would be an estimated 5–7 radiation-related cancers per 1000 men screened, and 6–13 cancers per 1000 women screened. Risk estimates are higher for women than for men whenever there is breast exposure (lung CT screening and coronary artery calcification screening).

**Table 1:** Estimates of the total lifetime risk of radiation-related cancer for various screening scenarios. The risks are expressed as the number of cancer cases expected in 1000 people

		Screening test, frequency and age at exposure				
Model type	Sex	Mammography every 3 years age 47–73	CT assessment of coronary artery calcification every 5 years age 45–70 males, age 55–70 females	CT colonography every 5 years age 55–70	Lung CT every year age 50–70	Total risk from all screening tests
ERR	Males	n.a.	1.4	2.6	3.0	7.0
	Females	0.6	2.3	2.0	8.0	12.8
EAR	Males	n.a.	0.6	2.7	1.4	4.7
	Females	0.3	0.7	1.9	2.9	5.8

n.a. – not applicable

ERR – excess relative risk

EAR – excess absolute risk

Benefits, where established, should outweigh the small risk of radiation-related cancer. For example, a decade of post-menopausal mammographic screening is estimated to prevent about one breast cancer death per 400 women screened.^11^ The first randomized trial of lung CT screening found that three annual screens reduced lung cancer mortality by 20% in heavy smokers,^12^ equivalent to about three lung cancer deaths prevented per 1000 screened. An important caveat though is that even if the benefits do outweigh the radiation risks at older screening ages this may not be the case for screening at younger ages because the radiation risks are higher but the absolute benefits are lower. The balance may also differ for higher risk sub-groups such as smokers or women with a family history of breast cancer. Situations such as these require detailed evaluation. The value of CT coronary artery calcification screening and CT colonography has not been established directly. Even small risks, therefore, could outweigh benefits.

The dose estimates for mammographic screening were based on national dose survey data from the UK.^6^ For the other screening examinations the doses were estimated using published screening protocols.^7–9^ These have been developed specifically to ensure low doses whilst maintaining the necessary image quality. It is important that protocols are optimized in this way and that quality control programmes are in place to monitor doses.

The use of ionising radiation in medical screening has increased in recent years. This raises legitimate health concerns. It is important to consider the potential risk of radiation-related cancer from screening and diagnostic imaging when setting public health recommendations for their use.

**Advisory Group on Ionising Radiation, Solid Cancer Risk Sub-group:**

**Chairman:** Professor Sir Nicholas Wald, London

**Members:** Dr Amy Berrington de González, Professor Bryn Bridges, Professor Doug Easton, Dr Mark Little, Dr Charles Stiller, Professor Martin Vessey

**Corresponding Members:** Dr Elisabeth Cardis, Professor Sarah Darby, Professor Leo Kinlen, Dr Kiyo Mabuchi, Professor Tony Swerdlow

**Secretariat:** Dr Colin Muirhead, Dr Richard Haylock

**HPA Representative:** Dr John Stather
